# Water displacement leg volumetry in clinical studies - A discussion of error sources

**DOI:** 10.1186/1471-2288-10-5

**Published:** 2010-01-13

**Authors:** Eberhard Rabe, Markus Stücker, Bertram Ottillinger

**Affiliations:** 1 Department of Dermatology of the University of Bonn, Sigmund-Freud-Strasse 25, 53229 Bonn, Germany; 2 Department of Dermatology of the University of Bochum, Gudrunstrasse 56, 44791 Bochum, Germany; 3 Ottillinger Life Sciences, Foehrenstrasse 12, 85649 Hofolding, Germany

## Abstract

**Background:**

Water displacement leg volumetry is a highly reproducible method, allowing the confirmation of efficacy of vasoactive substances. Nevertheless errors of its execution and the selection of unsuitable patients are likely to negatively affect the outcome of clinical studies in chronic venous insufficiency (CVI).

**Discussion:**

Placebo controlled double-blind drug studies in CVI were searched (Cochrane Review 2005, MedLine Search until December 2007) and assessed with regard to efficacy (volume reduction of the leg), patient characteristics, and potential methodological error sources. Almost every second study reported only small drug effects (≤ 30 mL volume reduction). As the most relevant error source the conduct of volumetry was identified. Because the practical use of available equipment varies, volume differences of more than 300 mL - which is a multifold of a potential treatment effect - have been reported between consecutive measurements. Other potential error sources were insufficient patient guidance or difficulties with the transition from the Widmer CVI classification to the CEAP (Clinical Etiological Anatomical Pathophysiological) grading.

**Summary:**

Patients should be properly diagnosed with CVI and selected for stable oedema and further clinical symptoms relevant for the specific study. Centres require a thorough training on the use of the volumeter and on patient guidance. Volumetry should be performed under constant conditions. The reproducibility of short term repeat measurements has to be ensured.

## Background

Various authors have considered water displacement leg volumetry as a gold standard or reference method for evaluating therapies working on the venous system of the lower extremities [1-3]. Optoelectronic methods building a three-dimensional model of the leg have advantages because they allow quicker measurements, but they require complicated machinery and are not more accurate or more reproducible than water displacement leg volumetry [2]. Simple measurements based on the frustrum method (i.e. modelling the leg as a section of a cone) are quick, but are often regarded as not very accurate [2].

Water displacement leg volumetry is based on a simple physical principle. If a leg is immersed into a container filled completely to a spout, the volume of overflowing water represents the volume of the leg, as far as it is immersed. The overflown water can be weighed or measured in a calibrated container. This simple principle allows static measurements in resting patients. More complicated variants have been developed to measure dynamic leg volume changes during exercise or rest. When describing their study equipment, most authors refer to a plethysmograph monitoring continuous volume changes developed by Thulesius et al. [4]. However, most studies have actually used modifications of this specific plethysmograph or devices only suitable for static measurements.

We noticed that studies in chronic venous insufficiency (CVI) using water displacement leg volumetry with the same or similar drugs had produced dissimilar results. As we assumed that the way of performing the study rather than lack of efficacy may have influenced the outcome [5-10] we performed a selective review of reports from clinical trials. Our aim was to elucidate potential error sources of static leg volumetry and to evaluate their importance for the study result. Based on our findings we give hints as to how the critical factors can be controlled when planning CVI studies with water displacement leg volumetry.

## Discussion

### Methods

We identified comparative clinical trials on drug therapies for CVI. We considered studies which were placebo controlled and described as double-blind. Patient populations were either characterised with the Widmer or with the CEAP (Clinical Etiological Anatomical Pathophysiological) classification. The studies evaluated static volume changes as opposed to dynamic plethysmometry, and used water displacement volumetry as opposed to optoelectronic methods. Starting with a Cochrane review on phlebotonics for venous insufficiency [11] and a MedLine search up to December 2007 with the keywords "foot volumetry" or "leg volumetry", we subsequently reviewed the abstracts and hand searched references in the studies selected this way. Studies with insufficient information on methodology and/or magnitude of effect were excluded. From these papers we extracted information on effect (reduction of leg volume), on patient characteristics, on the clinical setting, and on error sources of leg volumetry.

### Results

The Cochrane review contained n = 159 references and the MedLine search found n = 74 papers (n = 54 on leg volumetry and n = 61 on foot volumetry, with overlapping results). We reviewed n = 40 papers in full, where abstracts or descriptions in referring papers promised information on treatment effect and/or methodological aspects, and excluded n = 7, because these expectations were not met. Eleven publications (see Table 1) contained sufficient data for inclusion in our evaluation of treatment effects. We excluded the study by Burnand et al. [12], because it was planned to investigate skin and tissue oxygenation, not volume changes, and the study by Cesarone et al. [13], because it was not described as double-blind. In addition, both studies did not classify patients according to the Widmer or CEAP scales.

**Table 1:** Studies with venoactive drugs using water displacement leg/foot volumetry as an efficacy endpoint

First author, year of publ.	Patients	Centres	Drug/Reference (No. of patients with data)	Effect (volume)	Comments
**Casley-Smith 1988 **[33]	CVI Grade [I-]* III (Widmer) * not clear whether I-II was included	Monocentre, hospital. One study country (Australia)	Calcium dobesilate (n = 15), placebo (n = 15). Treatment duration 6 weeks	-85 mL vs. pbo	Change pre-post 4 mL (pbo) SE ca. 27 mL

**Rudofsky 1990 **[7]	CVI Grade I-II (Widmer)	Multicentre. One study country (Germany)	Ruscus + hesperidine-methylchalcone (n = 70), placebo (n = 71) Treatment duration 8 weeks	-47 mL vs. pbo (varicosis) -65 mL vs. pbo (postthrombotic syndrome)	Change pre-post ca. +30 mL (pbo) SE* ca. 6 mL

***Vanscheidt 2002a ***[10]	*CVI Grade I-II * *(Widmer)*	*Multicentre. * *One study country (Germany)*	*Ruscus extract (n = 77), placebo (n = 71) * *Treatment duration 12 weeks*	*-21 mL vs. pbo.*	*Change pre-post +6 mL (pbo)*

***Diebschlag 1994 ***[5]	*CVI Grade II (Widmer)*	*Monocentre. * *One study country (Germany)*	*Oxerutin 500 and 1000 mg (n = 2 × 20), * *placebo (n = 20). * *Treatment duration 12 weeks*	*-8 and -19 mL vs. pbo (500 and 1000 mg, resp.; corrected diff. vs. less affected leg)*	*Change pre-post ± 1 mL (pbo)* SE* ca. 1 mL

**Unkauf 1996 **[9]	CVI Grade II (Widmer)	Multicentre, outpatients. One study country (Germany)	Oxerutin (n = 64), placebo (n = 56); stockings in both groups. Treatment duration 12 weeks	-31 mL vs. pbo	Change pre-post -33 mL (pbo)

**Ihme 1996 **[28]	CVI Grade I-II (Widmer)	Monocentre, hospital. One study country (Germany)	Buckwheat herb tea (n = 36), placebo (n = 31). Treatment duration 3 months	-78 mL vs. pbo	Change pre-post +110 mL (pbo) SE* ca. 56 mL. Volumes determined by two different volumetric measurements

***Vanscheidt 2002b ***[32]	*CVI Grade C3-C5 * *(CEAP)*	*Multicentre, outpatients. * *Multinational (Germany, Austria, Switzerland)*	*Coumarin + troxerutin (n = 113), placebo (n = 113). * *Treatment duration 16 weeks*	*-30 mL vs. pbo*	*SE ca. 12 mL*

**Diehm 1996 **[6]	CVI mainly Grade I (Widmer)	Multicentre, hospital. Prob. one study country (Germany)	HCSE (n = 95), placebo (n = 46), compression (n = 99). Treatment duration 12 weeks	-54 mL vs. pbo	Change pre-post ca. +10 ± 15 mL (mean ± SE; pbo) and -47 ± 8 mL (compression)

***Diehm 2000 ****(not published, according to *[8]*)*	*CVI Grade II-III * *(Widmer)*	*Multicentre, hospital. * *Prob. one study country (Germany)*	*HCSE (n = 143), placebo (n = 70), compression (n = 142). * *Treatment duration 16 weeks*	*-20 mL vs. pbo*	Change pre-post ca. +2 ± 10 mL (mean ± SE* pbo) and -89 ± 10 mL (compression)

***Danielsson 2002 ***[14]	*CVI Grade C2-C4 * *(-C6) (CEAP)*	*Monocentre, hospital. * *One study country (Sweden)*	*Flavonoids (n = 48), placebo (n = 49). * *Treatment duration 2 months*	*+7.5 mL vs. pbo*	*Change pre-post +4 mL (pbo) * SE* ca. 4 mL. *Volumetry after 20 knee bends*

**Kiesewetter 2000 **[19]	CVI Grade I-II (Widmer)	Multicentre, outpatients. One study country (Germany)	Red vine leaf extract (360 mg: n = 86; 720 mg: n = 84), placebo (n = 87). Treatment duration 12 weeks	-76 and -100 g vs. pbo (360 and 720 mg, respectively)	Change pre-post ca. +34 g (pbo). SE* ca. 10 g

CVI: Chronic venous insufficiency; SE: Standard error; *: SE estimated from published data on group size and standard deviation; pbo: placebo; CEAP: Clinical Etiological Anatomical Pathophysiological CVI classification; HCSE: Horse chestnut seed extract

Further two studies included in our evaluation warrant comment: Danielsson et al. [14] examined foot volume not at rest but after 20 knee bends, so that the results are not fully comparable to the other studies. The study by Diebschlag et al. underestimates treatment effects compared to other studies because effects of therapy were calculated as the volume difference between the more severely and the less severely affected leg [5]. This was done to correct "for spontaneous changes in leg volume, related to variations in temperature, etc." As patients were not required to have strictly unilateral CVI, effects of active therapy on the less affected reference leg were subtracted during calculation, so that "these corrected volume changes are less pronounced than the absolute volume changes".

#### 1. Magnitude of Effect

The studies in Table 1 found a volume effect of therapy vs. placebo between +7.5 mL (i.e. a superiority of placebo) and -100 mL (i.e. a superiority of the active drug). To facilitate the detection of possible factors that might influence the magnitude of effect apart from the specific drug used, we divided the studies into two categories: Studies showing an only small to moderate effect of active drugs, which reduced foot/leg volume not at all or by up to 30 mL vs. placebo, and studies reporting a decrease in volume by more than 30 mL. The former group, which includes five of the eleven evaluated studies, is in italics in the table for illustrative purposes.

The cut-off of 30 mL has not been chosen arbitrarily. Marshall et al. have suggested that a volume reduction of 30-60 mL vs. placebo is a clinically relevant effect [15]. The authors estimate the total volume of lower leg subcutaneous tissues (epifascial space), the potential space where oedema may accumulate, at approximately 650 mL, with an actual mean oedema volume in CVI of 220 mL [15,16]. Thus, 220 mL is the highest mean volume that could be mobilised by a maximally effective therapy and a reduction of 30-60 mL corresponds to approximately 15-30% of this effect. An effect of this magnitude is probably clinically relevant, as elastic compression stockings, an accepted standard of care in CVI, reduced lower leg volumes by 33-89 mL in our panel of studies and thus to a similar extent (pre-post comparisons) [6,8,9].

A current guideline on how studies in CVI patients should be performed refers to the 30-60 mL border of clinical relevance, but in addition requires improvements in clinical symptoms and quality of life [17]. We did not evaluate the studies in our panel, whether they met this criterion, because our aim was not a meta-analysis on the efficacy of drugs used for CVI, but the detection of error sources in water displacement volumetry.

#### 2. Type of volumeter

Two major subtypes of volumeters are in use. The first and more common variant, shortly described in the Introduction, uses a container with an overflow spout. Water is filled into the container until water flows from the spout and the water flown over is discarded. Thereafter the patient lowers the limb into the container. The water which now flows from the spout is weighed or its volume is measured, indicating the volume of the limb lowered into the container.

The second variant measures the level of the water in the container, first before the patient lowers the limb into the container, and again with the limb in the container (or vice versa). From a calibration curve established with bodies of known volume, the rise or fall of water levels can be translated into volume changes.

Both types of volumeter possess a comparable precision in the 0.1-0.2% range for measurements of standard bodies and in the 0.1-1.0% range for repeated tests of patients' limbs [2,3,5,6,15,16,18,19]. In absolute values and in relation to total lower leg volumes of 2,200-3,200 mL, this corresponds to volumes between approximately 2 and 30 mL. A volume change of 30 mL is already at the border of clinical relevance as discussed above. Thus, we advise to test volumeters intended for clinical studies, to select an instrument with a high precision, and to optimise procedures until a reproducibility of short-term measurements in humans better than 20 mL is obtained. This is a realistic and achievable aim [5,15].

Furthermore, because oedema formation may be different in different regions of the lower leg, the height of the device has to be adequate for the study indication. For studies in venous patients the water level must be clearly above the pretibial region, where venous oedema typically accumulates.

#### 3. The method of leg volume measurements - equipment, procedures, and study centres

Studies in Table 1 and other publications on methodology [18,20-25] describe several error sources in leg volumetry. Table 2 presents an overview.

**Table 2:** Error sources in leg volumetry

**Methodology**
• Positioning of patient during resting and examination
• Room and water temperature
• Exclusion of superficial venous system with tourniquet
• Time of investigation
• Volumetry procedures
• Centres

**Indication/Patients**
• Cardiovascular co-morbidity or co-medication
• Other co-medication with volume effects
• Diagnosis and grade of CVI

CVI: Chronic venous insufficiency

##### 3.1 Positioning of patient during resting and examination

Changes in the patient's position during resting or before examination may result in a major change of leg volume. If patients rise from a supine to a standing position, the hydrostatic pressure rises to approx. 80 mmHg. This leads to a distension of the veins with an immediate increase of intravascular volume of approx. 500 mL [26]. If a sitting patient stands up, hydrostatic pressure and intravascular volume will change by approx. 30 mmHg and 190 mL, respectively. The volume increase may be higher in well-trained ("aerobic") persons due to their increased lower leg venous capacity compared to less trained persons [24]. Pannier could show with the Perometer method that the leg volume increases after the change from the supine to the standing position by app. 2.5%. A steady state is only reached after 10 minutes. These rapid changes are mainly due to an intravascular increase of blood volume [27].

In contrast to hydrostatic variations of intravascular volumes, which occur within minutes, the adaptation of extravascular volumes to a new postural equilibrium takes longer. As leg volumetry measures both intra- and extravascular volumes, experimental procedures should allow for this adaptation period.

Krijnen et al. observed that in CVI patients with a standing occupation the leg volume increased by approximately 80 mL over a full working day [23]. In healthy volunteers leg volumes reached a slightly smaller increase (51 ± 32 mL) during 30 minutes of standing [24].

Taking into account that CVI patients generally show a larger increase of leg volumes during standing due to an impaired integrity of their vascular walls, the major part of extravascular volume adaptation to a new limb position appears to take place within the first half hour. Thus we consider this time is a suitable and sufficient resting and adaptation time for patients before volumetry.

Water displacement volumeters generally require that the patient is sitting or standing. One point in favour of the standing position is the fact that this position imitates "the condition when venous hypertension is acting on the microcirculation and promoting damage to the tissue" [20]. However, with a duration of volumetry of approximately 15-30 minutes per patient [19,21], repeated measurements may drift to larger values (approx. +50 mL), if patients are examined in a standing position [24].

A further consideration supports volumetry in sitting patients: Due to both the immediate and the prolonged changes of intra- and extravascular leg volumes induced by postural changes, it appears important to have the patients move as little as possible between resting and volumetry. Because not all persons can tolerate a standing position over an adaptation period of 30 minutes [24], the patients should sit down to rest for not less than 30 minutes before volumetry, with the legs hanging down. For the examination the patients should ideally remain seated on the chair they sat during waiting and they should just lift their leg/s into the volumeter without moving around.

We discourage having patients rest with their legs crossed, though this has been described in one of the studies in Table 1 which found a large treatment effect [19]. Crossing legs impedes venous flow, similar to (though less pronounced than) a cuff. Data from a study using an inflatable cuff show that a cuff increases leg volumes by approx. 30 mL [1]. The effect of resting with the legs crossed is non-symmetrical, because it depends on which leg was the upper leg last before volumetry.

According to our findings one point has never been addressed - having patients rest with or without their shoes and socks/stockings. To avoid any compression, but also to reduce manipulation and extensive use of limb muscles during removal of those garments, shoes and socks/stockings should be removed before the waiting period starts and not directly before volumetry. This detail is a further confounder, if not described expressly in the study protocol and if study centres deal with this aspect differently.

##### 3.2 Room and water temperature

The clinical studies in Table 1 used water temperatures between 24 and 34°C. This appears reasonable: King found no noticeable difference in limb volumes at water temperatures of 20 and 35°C. More extreme temperatures of 5 and 45°C resulted in significant deviations of 1.4% from the normal temperature mean [22]. Thus changes of water temperature over the duration of the experiment will not unduly influence volumetry, and a check - but not a thermostatic control - of water temperature appears sufficient.

We found no data reporting that room temperature influences volumetry. However, room temperature should be controlled to an extent that patients neither feel cold nor sweat, because these mechanisms of thermoregulation will affect the distribution of blood flow between lower and superficial vessels of leg and foot and thus volumetry results.

##### 3.3 Exclusion of superficial venous system with tourniquet

In clinical practice, tourniquets are used in connection with volumetry to evaluate reflux times in superficial venous incompetence [13] or to predict the success of surgery in patients with varicosis [20]. Against this background, some centres may use tourniquets routinely in patients undergoing volumetry.

In volumetry studies evaluating the oedema reducing effect of venoactive drugs tourniquets have no place and we found no studies to the contrary. Here a tourniquet would introduce an artificial situation without clinical relevance. Centres should be educated to observe this detail, because habitual use of a tourniquet would introduce an easily avoidable bias into the study results.

##### 3.4 Time of investigation

Study protocols generally require patients to return for visits at approximately the same time of day to avoid the diurnal variability of leg volumes. Krijnen et al. found that over the day the leg volume of patients with major CVI increased by approximately 80 mL [23]. This corresponds to treatment effects of drugs in CVI, so that a bias introduced over non-standardised examination times could completely obliterate therapeutic effects.

An examination in the morning [5] may be of advantage, because the patients had less opportunity to participate in non-standardised activities over the day influencing leg volumes. On the other hand, oedema and leg volumes may have "settled" in the afternoon [19], i.e. change less over hours than in the morning [18], which decreases variability. We consider the exact time of examination of minor importance, if this time is standardised at all.

##### 3.5 Volumetry procedures

Practical use of a volumeter includes major sources of error. In one unpublished study, which used a volumeter with an overflow spout, one centre produced deviations larger than ± 20 ml between two repeated measurements (interval between measurements 5-10 minutes) in six of seven patients [Rabe, personal communication]. Some deviations were larger than 300 ml. Third repeat measurements were sometimes close to one of the original values, but as often somewhere in between or far outside the original range. Such differences are not explained by an imprecision of the instrument or variations over time. They indicate errors in performing volumetry and a profound misunderstanding of the procedures involved.

Three main error sources may be responsible: First, the containers may not have been fully filled before the first and/or second measurement, so that the overflow of water started when the limb was already partially immersed. This underestimates limb volumes. Second, water flown over during the initial filling of the container was not discarded, but remained in the container and was added to the water flown over during the measurement. This overestimates limb volumes. Third, the scales may have been calibrated belatedly while water was already running from the spout or without the collection container on the scale.

We advise that in absolute values, short term repeated measurements in patients should be within 10 to < 20 mL and deviations of 20 mL or more should lead to a further repetition and to a review of procedures.

##### 3.6 Centres

Centres may work with erroneous or varying procedures during volumetry, as stated above. So we expected that error sources and variability increase in multicentre and multinational studies and that those studies would less frequently find volume effects of active therapy which are > 30 mL. However, from the overview of studies in Table 1 we could not identify such an effect. Of four monocentre studies, two each found volume effects ≤ or > 30 mL, respectively, of seven multicentre studies three found a volume effect ≤ 30 mL, four an effect > 30 mL (*P *= 1.000; Fisher's exact test). Standard errors of pre-post changes in the placebo group were between 1 and 56 mL in the monocentre studies and between 6 and 15 mL in the multicentre studies.

We conclude that the error source centre can be overcome by teaching study staff in detail on the procedures of the specific volumeter. This is important, as technical details and procedures for volumeters vary. If centres have already experience with volumetry and are recruited for studies, it is likely that this experience has been gathered with volumeters and measurement procedures different from the study equipment.

Apart from education, performance of centres should be supervised closely and early. It is advisable to include volumetry at the screening visit and to monitor each single measurement (i.e. single values of repeat measurements) immediately either by electronic online forms or by faxed documentation pages. Centres with inexplicable deviations can be re-trained or closed, before they introduce major variability into the study.

The volume change during a run-in/wash-out period should be monitored. Changes depend on the study design, e.g. on whether compression and/or diuretics are instituted or ended. Ihme et al. found mean changes of up to 21 ml over a two-week placebo run-in phase [28]. In the mentioned unpublished study this change was between 71 and 151 g in one centre [Rabe, personal communication]. With hindsight, this low performing centre could have been identified and closed very early in the study. However, larger volume deviations during the run in phase could also be due to "unstable" oedema, for example CVI which is not fully stable or recent inflammation. In consequence oedema should be in a steady state before including a patient in a study.

#### 4. The patient - indication, co-diagnoses, patient selection, and patient characteristics

Vanscheidt et al. have published an international guideline describing how to test CVI drugs [17].

##### 4.1 Cardiac co-morbidity or co-medication

Lower limb oedema is a symptom of cardiac diseases. Oedema of cardiac origin show a considerable diurnal variation (164 ± 88 mL) [18] and are quickly influenced by concomitant drugs like diuretics. To avoid confounding of CVI related leg volumes, patients with cardiac co-morbidity should be strictly excluded from CVI studies.

##### 4.2 Other co-medication with volume effects

Drugs without primary cardiovascular (CV) action such as non-steroidal anti-inflammatory drugs (NSAIDs) may also influence limb oedema. They led to clinically manifest oedema in up to 3% of patients and increased mean body weight by up to 800 g [29,30]. Exclusion of this possible confounder and education of centres appear indicated.

##### 4.3 Diagnosis and grade of CVI

Vanscheidt et al. stress the point that clinical examination is not sufficient to ascertain a reliable diagnosis of CVI [17]. The disturbances of venous haemodynamics have to be shown by functional and imaging methods. Danielsson et al. observe that earlier studies in chronic venous disease (CVD) "have included patients with all kinds of symptoms from the lower leg, common for patients with CVD, but also present without CVD" [14].

The involvement of the superficial venous system, the deep venous system, or both should be proven with objective measures [14]. Venoactive drugs may possess a different efficacy on CVI of superficial and/or deep venous origin [7]. Accordingly, in- and exclusion criteria defining the extent and origin of CVI and tailoring the patient sample to the properties of the investigational drug are required. Otherwise the repetition of studies with one and the same drug is hazardous [6,8], because the selection of a comparable patient population is not assured.

We suspect that also the switch from the Widmer to the CEAP classification for CVI diagnosis could influence the results of repetitive studies.

The Widmer classification of CVI has been used for years especially in German speaking countries, the region where the majority of drug studies in CVI originates. Most centres that perform such studies are familiar with the Widmer classification and some still classify their patients according to it. The Widmer classification has been criticized, because it is based entirely on clinical, morphological criteria (dilated subcutaneous veins and skin changes) and does not take into account venous haemodynamics [17]. Variants of the classification also include information on oedema [31]) whereas in the original classification oedema is only a possible additional sign in all stages.

The CEAP classification includes aetiological, anatomical, and pathophysiological information and its construction has been validated. Current guidelines therefore recommend that patients for CVI studies should be classified according to the CEAP system [17]. Frequently only the "C" element of the CEAP score is used. In advanced stages there is little difference in information between Widmer-based classifications and the CEAP score, though the scores possess different numbers (Widmer Stages II correspond to CEAP Class 4, Widmer Stage III corresponds to CEAP Classes 5 and 6). Widmer Stage I defined only by corona phlebectatica has no clear counterpart in CEAP but may be mixed with C1. CEAP C3 has no clear counterpart in Widmer classification as there is no defined "Oedema Stage" but oedema can be present in Widmer Stages I - III.

It appears possible that a change of the CVI classification scheme can irritate investigators experienced with another scheme to an extent that they involuntarily and inadvertently include a different patient population. So the requirement to recruit patients with oedema and only mild skin changes (CEAP Grade 3-4a, i.e. Widmer Grades I-II) could induce investigators to recruit only Widmer Grade I patients and to exclude Widmer Grade II with more severe skin changes. Interestingly, the two studies in Table 1 which used the CEAP classification showed no relevant drug effect [14,32]. We will follow up this hypothesis when we report the mentioned unpublished study.

## Conclusions

Water displacement volumetry is a gold standard and reference method for evaluating lower limb volumes and the efficacy of venoactive drugs [1-3]. Nevertheless studies and experience show that it is easier to perform leg volumetry wrong than right. Error sources can introduce variations as large as drug effects. This affects sample size estimates and thus the success of a study, if statistical significance is missed. Lack of adherence to procedures defined in the study protocol affects the validity of the measurements or may result in the recruitment of a different patient sample from planned.

There are some possible errors from the patients' side. They may not strictly adhere to requirements on examination schedules or on body and limb positioning during waiting. Furthermore they may not inform the investigators of concomitant comorbidity or they may disregard instructions on impermissible concomitant medication. Accordingly investigators should carefully select patients with proven reliability regarding drug intake, accuracy of information provided, and appearance to scheduled examination dates and times. To achieve and maintain co-operation, it is vital that investigators inform patients of the detailed study procedures - at and between visits - and also of the "things that can go wrong", if these details are not observed.

Other error sources are within the investigators' responsibility. There are obvious deviations from the procedures described in the study protocol, like resting time and positioning, which introduce errors if not observed, but they usually should not reach a magnitude to completely invalidate study results. However, disregarding instructions on how to use the volumeter and on how to perform the examination could have this effect. We have seen deviating and varying volume measurements that can only be explained by a gross misconception of the evaluation procedure like calibrating devices or scales at wrong times or with wrong volumes. Some of these errors are caused by ordinary carelessness or by delegation of volumetry to inadequately trained staff. In addition and probably more importantly, there is a variety of volumeters which use the same physical principle - water displacement - but apply different methods of reading out the results: Water level, weight of displaced water, volume of displaced water, null value before lowering the limb into the volumeter or after removing it. Thus centres using volumetry in clinical practice or for studies may be familiar with the concept and a certain type of volumeter, but not with a specific device selected for a specific study. We see a danger that out of habit centres may apply their procedures for their usual volumeter to a different device. This could result in gross and irreversible errors.

It is the responsibility of the study sponsors to assure training of investigators and staff. Volumetry should not only be presented during an investigators' meeting. It should be trained in person in the centres in the presence and with the active participation of the relevant staff. Sponsors should make sure whether centres are experienced with other volumeters or other procedures (e.g. regarding the position of the patient during volumetry or the use of a tourniquet) - and if this is the case, to specifically address such differences. Centres experienced with other types of volumeter require as close supervision during the study as centres that use volumetry for the first time and thus are not influenced by former habits.

Furthermore, sponsors should ascertain that investigators understand that the situation in a clinical study differs from routine practice and requires sufficient capacity as to time and staff. This capacity is necessary to identify the right patients, to guide and supervise them, and to perform volumetry correctly.

We assume that a further highly important error source within the sponsor's responsibility is the definition of study patients and the supply of adequate instructions to investigators how to identify exactly these patients. The switch from the Widmer classification of CVI to the CEAP classification with an accepted transition rule [17] may result in the selection of a different patient population. The only two studies of our evaluation which used the CEAP classification showed no relevant drug effect [14,32].

## Summary

Successful studies with venoactive drugs in CVI using lower limb volumetry as an efficacy endpoint require a multifold approach.

• The patients should not only be recruited according to CEAP grades, but investigators should also receive clear guidance as to relevant accompanying clinical symptoms. The exact CVI severity grade to be selected for the study depends on the drug and its pharmacological mode of action.

• Patients with cardiac co-morbidity and patients using drugs with effects on the leg volume (like NSAIDs) should be excluded from CVI studies.

• Detailed written and verbal instructions to investigators on how to perform volumetry are essential. This includes the task to explain body and limb positioning during waiting to the patients.

• Volumeter and procedures should be optimised until a reproducibility of short term repeat measurements in humans better than 20 mL is obtained.

• Only trained and certified staff should perform volumetry. Close and prompt supervision throughout the study should be available.

Failure of studies due to unidentified error sources and inadequate performance is not only an issue of misspending budgets. It is rather an ethical issue, because patients have in good faith taken on themselves the risks or discomforts of a study, with the aim to help in the collection of conclusive data. We hope that our discussion of error sources in studies using leg volumetry helps in avoiding some of these pitfalls in future studies.

## Abbreviations

CEAP: Clinical Etiological Anatomical Pathophysiological; CV: cardiovascular; CVD: cardiovascular disease; CVI: chronic venous insufficiency; HCSE: horse chestnut seed extract; NSAID: non-steroidal anti-inflammatory drug; pbo: placebo; SE: standard error

## Competing interests

ER, MS: There are no competing interests concerning this paper. This includes financial and nonfinancial competing interests. BO is an independent consultant for the medical and pharmaceutical industry. He has received honoraria for the preparation of this manuscript.

## Authors' contributions

ER carried out literature research, analysis and interpretation of data, and drafted and corrected the manuscript. MS contributed to literature research, analysis and interpretation of data, and to corrections in the manuscript. BO participated in the conception of this project, contributed to writing the manuscript, and coordinated the authors' comments. All authors read and approved the final manuscript.

## Pre-publication history

The pre-publication history for this paper can be accessed here:

http://www.biomedcentral.com/1471-2288/10/5/prepub
